# Endoscopic retrograde cholangiopancreatography versus laparoscopic exploration for common bile duct stones in post-cholecystectomy patients: a retrospective study

**DOI:** 10.18632/oncotarget.18839

**Published:** 2017-06-27

**Authors:** Xiaohong Wang, Chenguang Dai, Zhonghua Jiang, Lili Zhao, Min Wang, Limei Ma, Xueming Tan, Li Liu, Xiang Wang, Zhining Fan

**Affiliations:** ^1^ Department of Digestive Endoscopy and Medical Center for Digestive Diseases, The Second Affiliated Hospital of Nanjing Medical University, Nanjing, Jiangsu 210000, China; ^2^ Department of Gastroenterology, The Second Affiliated Hospital of Xuzhou Medical College, Xuzhou, Jiangsu 221006, China; ^3^ Digestive Endoscopy Center, The First Affiliated Hospital with Nanjing Medical University, Nanjing, Jiangsu 210029, China; ^4^ Department of Gastroenterology, The First People’s Hospital of Yancheng, Yancheng, Jiangsu 224006, China

**Keywords:** ERCP (endoscopic retrograde cholangiopancreatography), LCBDE (laparoscopic common bile duct exploration), common bile duct, post-cholecystectomy, retrospective study

## Abstract

**Background and Objective:**

Common bile duct (CBD) stones are common in patients even after cholecystectomy. Besides endoscopic retrograde cholangiography (ERCP), laparoscopic common bile duct exploration (LCBDE) is also applied. This study aims to compare clinical indications, therapeutic benefits and complications for these two managements.

**Methods:**

From October 2012 to February 2015, 1072 consecutive patients were diagnosed as choledocholithiasis in our single hospital. Post-cholecystectomy patients who underwent ERCP or LCBDE were included. Clinical data were analyzed, such as success rate, complications, procedure duration, postoperative hospital stay, total cost and recurrence of ductal stones. Prior ERCP, previous biliary anatomic alteration surgeries and lost to follow up were the excluding criteria.

**Results:**

141 patients were included according to the criteria, and 87 cases underwent ERCP and 54 cases underwent LCBDE. Age and sex distribution of patients were comparable between the two groups. The success rate for CBD stones clearance was 97.7% in the ERCP group, compared with 87.0% in the LCBDE group (*p*=0.03). The mean procedure duration was also significantly shorter in ERCP group (52.0±15.8 vs. 102.9±40.1 min; *p*<0.001). Postoperative hospital stay was similar (5.5±2.6 vs. 5.9±2.3 days; *p*=0.40). And no significant difference for postoperative complications (3.4% vs. 11.1%; *p*=0.15), total cost ($3787.1±1061.5 vs. $3983.54±1257.1, *p*=0.32), and the rate of bile duct stones recurrence (6.9% vs. 7.4%, *p*=1.00).

**Conclusions:**

For clearing CBD stones in patients after cholecystectomy, ERCP was more efficient and might be the first choice, while LCBDE might be beneficial for patients with large stones.

## INTRODUCTION

10∼18% of patients undergoing cholecystectomy for gallstones have complicating common bile duct (CBD) stones [1]. Choledocholithiasis represents a prevalent condition even in patients after cholecystectomy. Clinical Management for CBD stones includes open CBD exploration, endoscopic retrograde cholangiopancreatography (ERCP) and laparoscopic CBD exploration (LCBDE). With the development of micro-invasive techniques, open CBD exploration is sidelined only when the other techniques are ineffective or unavailable [2]. As a primary strategy for choledocholithiasis, ERCP is a well-established technique and has achieved great success since 1974 [2, 3]. Recently, several studies have shown that LCBDE might also be an effective intervention for CBD stones [4, 5]. Zhu *et al.* [6] reported that LCBDE was successfully used for 11 patients with choledocholithiasis after cholecystectomy. As reported [7], for preoperatively known choledocholithiasis, 86% of clinicians suggested ERCP; while for stones discovered intraoperatively, 30% selected LCBDE. After cholecystectomy, patients commonly had biliary strictures and abdominal adhesions, which might hinder further surgical approaches for CBD stones. But few studies have compared clinical superiority of the two methods for such patients. This retrospective study tried to compare clinical efficacy between ERCP and LCBDE for patients with CBD stones after cholecystectomy, including successful rates, complications, procedure time, hospitalization, cost and undesired recurrence.

## RESULTS

### Patient characteristics

**Figure 1:**
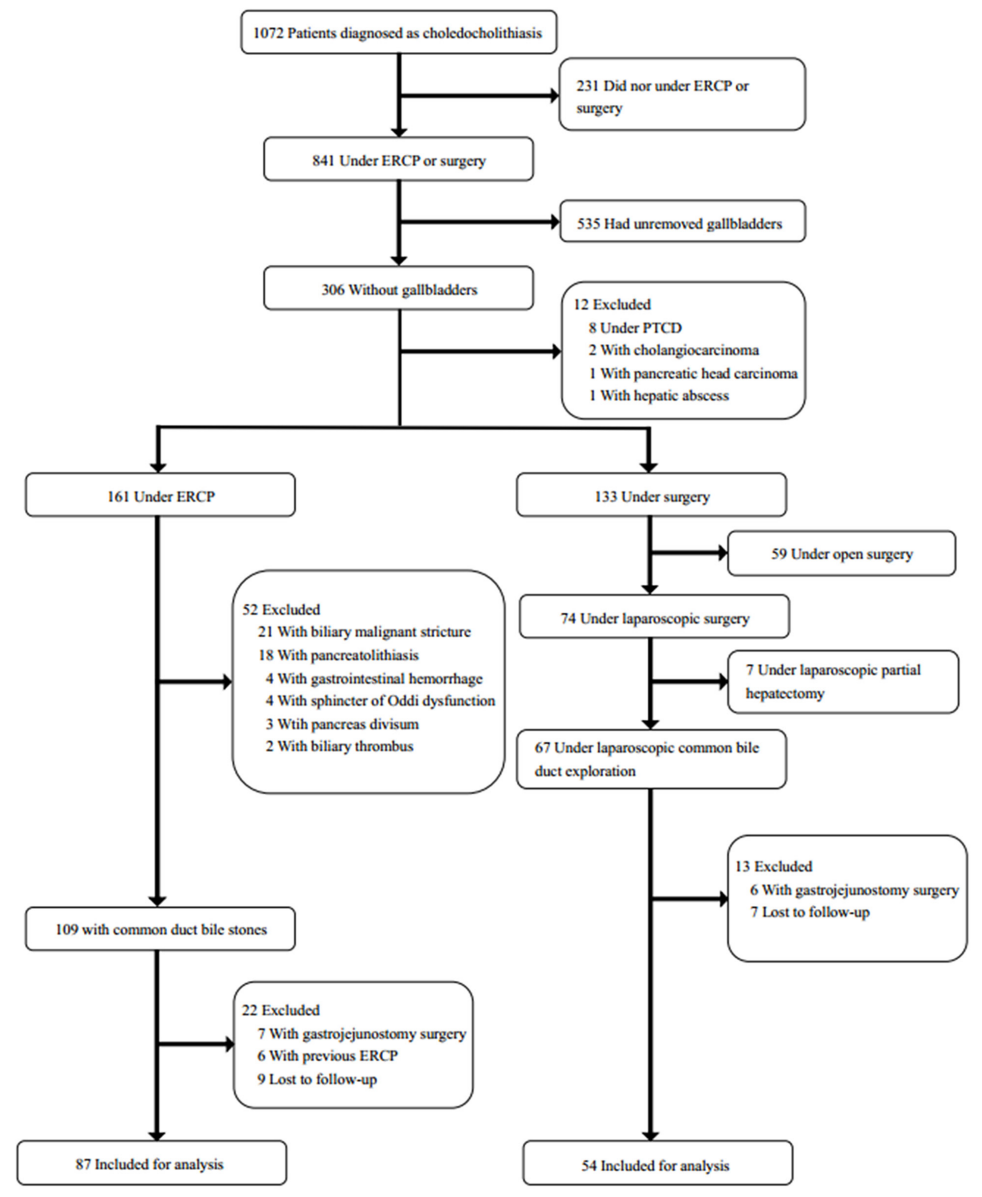
Flow chart

Between October 2012 and February 2015, a total of 1072 patients were referred to our hospital due to the primary diagnosis of CBD stones. And their stones were confirmed by MRCP, CT or abdominal ultrasonography. 231 patients recovered from the disease in conservative management, and 535 received laparoscopic cholecystectomy at the same time. In the other 306 patients, 161 patients were treated by ERCP and 67 patients were under LCBDE. For these 306 patients without gallbladders, the shortest and longest time span were respectively 1 month and 173 months. The mode time was 45 months. The median was 56 months. The mean was 62±38 months (standard deviation). In ERCP group, 65 cases were excluded due to biliary malignant stricture (21 cases), pancreatolithiasis (18 cases), gastrojejunostomy surgery (7 cases), previous ERCP (6 cases), gastrointestinal hemorrhage (4 cases), SOD (4 cases), pancreas divisum (3 cases) and biliary thrombus (2 cases). In LCBDE group, 6 cases were excluded because of previous gastrojejunostomy surgery. During the follow-up, 9 and 7 cases lost in ERCP group and LCBDE group respectively. Finally, 141 patients were eligible for analysis, 87 in ERCP group and 54 in LCBDE group (Figure 1).

Patients in this two groups were similar in terms of age (57.3 ± 15.4 VS 59.5 ± 13.3, *p*=0.38), sex distribution and BMI. Upper abdominal pain was the predominant clinical symptom for over 80% patients of both groups. There were no differences in other symptoms (fever, jaundice and pancreatitis) and coexisting disorders. ALT, AST, AKP and γ-GT levels were high, but not significantly different between the two groups. Serum bilirubin was slightly elevated in both groups (Table 1).

**Table 1:** Demographics and preoperative clinical characteristics of the patients

	Patients under ERCP (n=87)	Patients under LCBDE (n=54)	P value
Age, years (mean±SD)	57.3±15.4	59.5±13.3	0.38
Sex, male/female	36/51	22/32	0.94
Body-mass index, kg/m^2^ (mean±SD)	23.8±3.2	24.0±3.3	0.77
Symptoms, no. (%)			
Upper abdominal pain	76(87.4%)	51(94.4%)	0.17
Fever	3(3.4%)	7(13.0%)	0.07
Jaundice	15(17.2%)	12(22.2%)	0.47
Pancreatitis	3(3.4%)	1(1.9%)	0.97
Coexisting disorders, no. (%)			
Hypertension	21(24.1%)	11(20.4%)	0.60
Diabetes	8(9.2%)	6(11.1%)	0.71
Others †	7(8.0%)	4(7.4%)	1.00
More than 2 disorders	7(8.0%)	9(16.7%)	0.12
Method for cholecystectomy, no. (%)			0.70
Laparoscopic cholecystectomy	39(44.8%)	26(48.1%)	
Open operation	48(55.2%)	28(51.9%)	
Preoperative imaging examination ‡, no. (%)			0.02
Type-B ultrasound	21(24.1%)	25(46.3%)	
CT	14(16.1%)	4(7.4%)	
MRCP	52(59.8%)	25(46.3%)	
Preoperative laboratory test §(mean±SD)			
WBC, *10^9 cells/L	8.5±2.9	8.0±2.6	0.36
TBIL, μmol/L	32.2±42.3	28.9±39.8	0.65
DBIL, μmol/L	17.4±25.9	16.1±28.5	0.78
ALT, U/L	176.0±204.8	126.2±174.3	0.14
AST, U/L	128.4±159.5	88.4±135.3	0.13
AKP, U/L	245.6±202.4	210.9±193.8	0.32
γ-GGT, U/L	427.9±504.5	359.8±416.0	0.41
PT, s	11.5±1.7	11.5±1.0	0.80

† Other coexisting disorders included coronary heart disease, atrial fibrillation, asthma, hepatitis B, anemia and cataract.

‡ One in these three examination was successfully applied for diagnosing in different patients.

§ The normal range: WBC, 3.50-9.50*10^9 cells/L; TBIL, 5.1-19.0 μmol/L; DBIL, 0.0-6.8 μmol/L; ALT, 7-50 U/L; AST, 13-40 U/L; AKP, 30-120 U/L; γ-GGT, 7-60 U/L; PT, 11±3 s.

### Procedure-related outcomes

Both stone number and size were similar in the two groups (Table 2). 85 of 87 patients in the ERCP group had their CBD stones cleared at the first intervention. 2 cases failed. One with a large CBD stone could not be extracted by ERCP, then implanted with two biliary stents instead. For another patient, retained stone was found via ENBD cholangiography 5 days later, and then successfully removed by a second ERCP. Biliary strictures were found in 5 cases during cholangiography and all were successfully treated by balloon dilatation.

**Table 2:** Procedure-related clinical characteristics of the patients

	Patients under ERCP (n=87)	Patients under LCBDE (n=54)	P value
Procedure time, min (mean±SD)	52.0±15.8	102.9±40.1	<0.001
Stone number, no. (%)			0.34
1	41(47.1%)	21(38.9%)	
≧2	46(52.9%)	33(61.1%)	
Stone size, cm (mean±SD)	1.08±0.55	1.19+0.59	0.25
Intraoperative complications, no. (%)	0	0	/
Stone complete clearing, no. (%)	85(97.7%)	47(87.0%)	0.03
Postoperative complication †, no. (%)	3(3.4%)	6(11.1%)	0.15
Discharge with drainage tube, no. (%)			<0.001
Nasobiliary drainage	87(100%)	/	
T-tube	/	50(92.6%)	
Intra-ERCP			
EST, no. (%)	55(63.2%)	/	
EST length, cm (mean±SD)	0.5±0.2	/	
Balloon dilation, no. (%)	57(65.5%)	/	
ENBD, no. (%)	87(100%)	/	
Biliary stent, no. (%)	2(2.3%)	/	
Intra-LCBDE			
Choledochotomy, no. (%)	/	54(100%)	
Choledochoscopic exploration, no. (%)	/	54(100%)	
Abdominal drainage tube, no. (%)	/	53(98.1%)	

† Complications of the patients under ERCP were all post-ERCP pancreatitis.

Complications of the patients under LCBDE were 3 biliary infection, 2 biliary benign structure and 1 postoperative intestinal obstruction respectively.

In the LCBDE group, abdominal adhesion was found in all cases, which increased the difficulty for clearing the stones. At the first intervention, complete clearance was in 47 of the 54 patients. 3 failed cases were due to impacted stones in the distal bile duct, which were removed by ERCP. Retained stones were found in 4 patients during T-tube cholangiography one month later, and then were retreated by choledochoscope combined with micro-blasting lithotripsy. There were no cases of conversion to open surgery. Overall, the success rate in the ERCP group was higher than the LCBDE group (97.7% vs. 87.0 %; *p*=0.03) (Table 2).

There was no report about intraoperative complications during ERCP or LCBDE. The postoperative complication rate was lower in ERCP group, but there was no significant difference between two groups (3.4% vs. 11.1 %; *p*=0.15). In the ERCP group, 3 patients had post-ERCP pancreatitis and were managed conservatively. Postoperative complications occurred in 6 patients in the LCBDE group. 3 patients had cholangitis and were cured with antibiotics. 2 patients had biliary benign stricture and were successfully managed through balloon dilatation by ERCP. One patient experienced postoperative intestinal obstruction and was treated conservatively (Table 2).

The mean duration of LCBDE was 102.9±40.1min, which was significantly longer than ERCP (50.2±15.8min, *p*<0.001). The average postoperative stay was similar for both groups (ERCP 5.2 ±2.6 days versus LCBDE 5.9 ±2.3 days; *p* =0.40) (Table 2). Total costs were also similar for the ERCP and the LCBDE treatment ($3787.1±1061.5 vs $3983.54±1257.1, *p*=0.32) (Table 3).

**Table 3:** Postoperative and follow-up clinical characteristics of the patients

	Patients under ERCP (n=87)	Patients under LCBDE (n=54)	P value
Abdominal drainage tube removing time †, day (mean±SD)	/	4.2±1.9	
T-tube removing time ‡, day (mean±SD)	/	75.6±32.3	
Postoperative hospitalization time, day (mean±SD)	5.5±2.6	5.9±2.3	0.40
Total cost, $ (mean±SD)	3787.1±1061.5	3983.54±1257.1	0.32
Lost to follow-up, no. (%)	9(10.3%)	7(13.0%)	0.63
Follow-up time, month (mean±SD)	27.8±4.0	26.5±3.8	0.74
Recurrence §, no. (%)	6(6.9%)	4(7.4%)	1.00
Recurrence time ¶, month (mean rank)	11.3	11.8	0.81

† All abdominal drainage tubes were removed before discharge. The time was calculated from operation day.

‡ The time was calculated from operation day.

§ There were 6 patients under ERCP and 4 patients under LCBDE referring to hospital due to recurrent choledocholithiasis. And 1 patient in each group had 2 recurrence respectively.

¶ Mann-Whitney test was used for person-time.

### Patient follow-up

Follow-up was continued until March 2016. There were 9 patients lost to follow-up in ERCP group (9.4%) and 7 in LCBDE group (11.4%), which were not included for analysis. All the lost follow-up cases were successful for clinical treatment. Median follow-up was 27.8 months in ERCP group and 26.5 months in LCBDE group, respectively. 6 patients (6.9%) had recurrent bile duct stones in ERCP group and 4 patients (7.4%) in LCBDE group. One patient in each group had twice recurrence. The median recurrence time was 11.3 months in ERCP group and 11.8 months in LCBDE group, respectively. But there is no significant difference in recurrence rate or time. One recurrent case in LCBDE group was treated by open surgery. Others were managed by ERCP (7 cases) or drug therapy (2 cases).

## DISCUSSION

Gallstones is a common disease with a morbidity of approximately 15% [8]. Even after cholecystectomy, CBD stones would relapse. With anatomical alteration, cholecystectomy might result in biliary strictures and abdominal adhesions, which increased the difficulty of further clinical approach. In this study, 5 cases had biliary strictures in ERCP group, while abdominal adhesion was found in all cases of LCBDE group. The aims of this study was to investigate the clinical efficacy and superiority of ERCP and LCBDE for CBD stones in post-cholecystectomy patients.

In previous studies, CBD clearance rate of ERCP was 95%-97% [9, 10]^9, 10^in experienced hands [9, 10]. No difference existed for preoperative ERCP or postoperative ERCP procedure [11]. Similarly, our results demonstrated that success rate was a bit higher by ERCP than LCBDE (97.7% VS 87.0%, *p*=0.03). For ERCP, procedural failure was usually due to large and impacted stones, postsurgical gastrointestinal anatomic variations, duodenal diverticulum, or CBD strictures, which was also shown in this study. One failed cases was diagnosed with large stones in the common bile duct, which is commonly considered as difficult bile duct stones and limits safe extraction [12]. For large or difficult CBD stones, endoscopic biliary stenting is an effective strategy, which could facilitate bile drainage and prevent stone impaction or cholangitis before the surgical intervention or a second ERCP attempt [13]. Instead of stone extraction, patient’s symptoms relieved after implantation with two biliary stents. In another case unexpected retained stones were found by nasobiliary cholangiography 5 days later. Campagnacci R *et al.* [14] and Naumowicz E *et al.* [15] reported the retained stones percentage after ERCP was 9% and 13.5% respectively. In our cohort, retained stones appeared only in 1.1% (1/87) of patients after first ERCP, lower than these studies. Retained stone was removed easily by the second ERCP.

According to randomized controlled trials, successful laparoscopic CBD stone clearance was 75–100 % [4, 5, 16, 17]. The rate was also consistent in our study (80.0%). Common failure reasons were impacted stones or retained stones. Tinoco *et al* [18] showed 1.5% retained stones were found in 481 LCBDE, lower than our results (7.4%, 4/54). Most of the cases in our study had no intraoperative cholangiography in LCBDE, which might have influenced the detection for stones. It’s difficult for LCBDE to deal with the impacted stone, especially in distal bile duct. For ERCP, sphincterotomy could be applied to extract impacted stones from the bile duct. All 3 casee with impacted stones were successfully treated by ERCP. Choledochoscope combined with micro-blasting lithotripsy via T-tube was another potential approach for impacted stones and retained stones after failed LCBDE.

In our study, therapeutic efficacy was better by ERCP. Previous random control trials reported that the clearance rate between ERCP+LC with LCBDE +LC was similar [4, 5, 16, 17]. LCBDE+LC is one stage procedure, while the ERCP+LC is commonly applied in the two stages. ERCP is performed within 4 weeks after LC. Our study focused on the patients after cholecystectomy, and most cases were treated by ERCP or LCBDE at one year after cholecystectomy. Post-surgical anatomic alteration might hinder clinical performance of LCBDE. As aforementioned, biliary stricture was found for both groups, and abdominal adhesion was common in all LCBDE cases. All these increased the difficulty of extracting stones. Procedural duration was longer for LCBDE due to adhesion dissection (102.9±40.1minutes), while ERCP took 50.2±15.8 minutes as usual. All cases with biliary benign stricture were cured by balloon dilatation under ERCP [19].

In our study, there was no significantly difference for complications, while ERCP group looked lower than LCBDE group (3.4% vs 11.1 %). The ERCP complications include pancreatitis, hemorrhage, cholangitis, duodenal perforation and mortality [20–22]. Due to the cohort limit, ERCP complication incidence was lower than previous retrospective studies 7.92% ∼ 11% [17, 20]. Experienced endoscopists were also important for preventing undesired complications. Only 3 patients had post-ERCP pancreatitis and were managed conservatively.

For LCBDE, the complication rate was similar as previous reports (9.5%) [21]. postoperative infection happened in three cases. Two patients appeared with biliary benign strictures two months after LCBDE, then were treated by ERCP as recommended [19]. One postoperative intestinal obstruction was reported due to postoperative gastrointestinal tract dysfunction, which was common in surgical patients. No bile leakage was observed in this study, which was common for LCBDE [21, 23]. Compared with ERCP, LCBDE patients need to carry with T-Tube for 75.6±32.3 days, which might greatly affect their life quality.

Postoperative hospitalization and cost was also similar for both groups. ERCP and LCBDE group respectively took 5.5±2.6 and 5.9±2.3 days. It was mainly influenced by the complications. The longest case was 12 days in ERCP group due to postoperative pancreatitis, while 13 days for postoperative cholangitis in LCBDE group. The total cost was approximate in our study. Cost was affected by many factors, such as hospital style, operative time, postoperative hospitalization and complications. This study just included patients from our single hospital. Treatment cost might vary in different regions, which might influence the choice of patients and health care providers.

Follow-up results indicated that recurrence rate was similar in the two groups. There were 6 (6.9%) patients in ERCP group and 4 (7.4%) patients in LCBDE group. Previous reported showed that the recurrent CBD stone occurred in 7.9% the patients after LCBDE [24]. The median time of recurrence was 11.3 months in ERCP group and 11.8months in LCBDE group after the operation. Most cases (70%, 7/10) were treated by ERCP, due to its minimally invasive and repeatable characteristics.

In conclusion, our results confirmed that for patients with CBD stones after cholecystectomy, ERCP was recommended due to its clinical efficiency. There was no difference for postoperative complication, hospitalization and recurrence. Without T-tube, ERCP patients also experienced the better life. As the retrospective study, lack of randomization might cause undesired selection bias, though exclusion criterion was set to reduce the bias. And sample size limits the final conclusion in this study. The randomized controlled trials with large cohort will be designed for further validating our analysis results.

## MATERIALS AND METHODS

### Study design and population

A retrospective study was conducted including all consecutive patients who presented with CBD stones between October 2012 and February 2015 from our single hospital in China. Data were collected from the patients’ medical records. Patients were included for analysis when they met the following criteria: (1) CBD stones confirmed by Magnetic resonance cholangiopancreatography (MRCP), computed tomography (CT) and/or abdominal ultrasonography; (2) Medical history of cholecystectomy. The exclusion criteria was: (1) no ERCP or surgery in this course; (2) unremoved gallbladders;(3) pancreatolithiasis, sphincter of Oddi dysfunction (SOD), pancreas divisum, biliary thrombus or gastrointestinal hemorrhage; (4) other pancreatic or biliary malignant diseases; (5) open surgery; (6) partial hepatectomy at the same time; (7) history of LCBDE or ERCP for recurrent CBD stones; (8) history of biliary diversion, Billroth II surgery or Roux-en-Y surgery.

### Endoscopic technique

ERCP was performed by two experienced endoscopists who treat more than 300 cases every year. The patients were under sedation anesthesia. After wire-guided assisted cannulation, cholangiography confirmed the existence of CBD stones. Stones were removed by the extraction basket or balloon, while sphincterotomy, balloon dilation or mechanical lithotripsy was applied if necessary. A re-check cholangiograph was recorded for complete clearance. An endoscopic nasobiliary drainage (ENBD) tube was inserted routinely. Cholangiography was performed again via the ENBD tube 2-5 days later. If no CBD stones were retained, ENBD tube would be removed.

### Laparoscopic surgery procedure

LCBDE was performed by surgeons from Biliary Surgery Department. The operation was carried out routinely under general anesthesia. After inserting the Trocars and establishing pneumoperitoneum, laparoscope was used to explore the abdominal cavity firstly. Then the adhesion surrounding the CBD was separated carefully. After choledochotomy, a flexible choledochoscope was inserted into common bile duct to identify stones. The stones were removed by choledocholith pliers or basket. A rechecking choledochoscopy was applied to ensure CBD clearance. A T-tube was then placed in the CBD via the incision. While surgical closure of the abdominal incision, one abdominal drainage tube was placed along the gallbladder forssa commonly. The tube would be removed when the drainage appeared no abnormalities. Cholangiography through T-tube was usually performed one month later, and T-tube was removed if no CBD stones were identified. For retained stones, T-tube assisted choledochoscope or ERCP would be applied.

### Patient follow-up

Follow-up data was collected as the study design. Patients were surveyed respectively one month, three months and six months after their discharge. Postoperative complications, life changes, recurrence and any related concerns were recorded. For LCBDE, T-tube removing time, recurrence frequency and time were recorded.

### Outcome and statistical analysis

Demographics and preoperative characteristics including age, sex, body-mass index (BMI), clinical symptoms, coexisting disorders and laboratory tests were reviewed for accessing the comparability. Procedure-related, postoperative and follow-up clinical results were compared, including procedure time, stone number and size, complete clearance, complications, hospitalization time, total cost, recurrence frequency and time.

Statistical analysis was performed using the SPSS statistics 19.0. Continuous variables were tested using Student’s t test or nonparametric test (Mann-Whitney test) when appropriate. And data were reported as means with standard deviation. Categorical variables were described as counts and percentages and were compared using Chi-square test or Fisher’s exact test. All *p* values were two-sided and *p*<0.05 was considered statistically significant.

## Abbreviations

CBD: common bile duct
ERCP: endoscopic retrograde cholangiography
LCBDE: laparoscopic common bile duct exploration
MRCP: magnetic resonance cholangiopancreatography
CT: computed tomography
SOD: sphincter of Oddi dysfunction
ENBD: endoscopic nasobiliary drainage
BMI: body-mass index
